# Terrestrial and Airborne Laser Scanning Dataset of Trees in the Shivalik Range, India with Field Measurements and Leaf–Wood Classifications

**DOI:** 10.1038/s41597-026-06674-w

**Published:** 2026-02-11

**Authors:** Moonis Ali, Apratim Biswas, Anna Iglseder, Vinod Kumar, Shant Kumar, Sandeep Gupta, Markus Hollaus, Norbert Pfeifer, Bharat Lohani

**Affiliations:** 1 Department of Civil Engineering, Indian Institute of Technology Kanpur, Kanpur, 208016 UP India; 2 Department of Geodesy and Geoinformation, TU Wien, Vienna, Austria; 3 Haryana Forest Department, Panchkula, Haryana India; 4 Institute of Environmental Studies, Kurukshetra University, Kurukshetra, India

**Keywords:** Forestry, Forest ecology

## Abstract

Annotated datasets are essential for training and evaluating machine learning models in forest ecology. This dataset provides high-resolution, annotated LiDAR point clouds of 674 individual trees from 12 forest plots in the Shivalik Range of northern Haryana, India, representing 24 species. Data were acquired using Terrestrial Laser Scanning (TLS) and Airborne Laser Scanning (ALS), include field-measured attributes such as species identity and Diameter at Breast Height (DBH), and terrestrial and aerial RGB imagery. TLS point clouds were georeferenced and co-registered with centimetre-level accuracy, enabling precise integration with ALS data. The dataset includes segmented individual trees and wood–leaf classifications, suitable for applications such as tree morphology analysis, biomass estimation, and species classification. To support benchmarking, outputs from established classification algorithms (LeWoS, TLSeparation, CANUPO, and Random Forest) are included. As one of the first open-access LiDAR datasets from Indian tropical forests, it provides critical reference data for developing and validating forest structure models. It can also aid biomass mapping efforts in support of large-scale missions such as NASA-ISRO’s NISAR and ESA’s BIOMASS.

## Background & Summary

Forests play a critical role in regulating global climate, maintaining biodiversity, and providing essential resources such as timber, fuelwood, food, and medicinal plants^1–3^. Understanding the forest structure and dynamics at the individual tree level is crucial for effective forest management, biodiversity conservation, and climate change mitigation^4^. Traditionally, individual tree assessments have relied on field-based measurements, which are often labour-intensive, time-consuming, and spatially restrictive, limiting their applicability for large-scale forest monitoring^4,5^.

The rise of remote sensing technologies has revolutionised forest assessment by enabling efficient, scalable, and cost-effective data collection^6^. Technologies like LiDAR, combined with advances in computational power, have made it possible to derive detailed structural parameters—such as tree height, crown dimensions, and volume—at high spatial resolution up to the individual tree level^7–17^. These parameters serve as critical inputs to models that estimate key forest attributes, including Above Ground Biomass (AGB) and carbon content^18,19^, supporting programs such as REDD + (Reducing Emissions from Deforestation and Forest Degradation)^20,21^.

Terrestrial Laser Scanning (TLS) and Airborne Laser Scanning (ALS) have emerged as key tools for acquiring high-resolution three-dimensional (3D) representations of forest ecosystems. TLS are ground-based scanners, capturing detailed structural information of individual trees, including stems and branches. ALS, on the other hand, collects data from airborne platforms, typically aircrafts or UAVs, allowing broader coverage across large forested areas. These technologies enable accurate extraction of forest metrics such as tree height and diameter at breast height (DBH)^17,22–30^, crown dimensions^31,32^, basal area^23^ and wood volume or biomass^17,33–38^, with high accuracy and efficiency.

While TLS provides fine-grained details suitable for small forest plots, it is limited by slower data acquisition, restricted spatial extent and occlusion effect caused by lower branches, surrounding trees and understory^39^. ALS, conversely, supports landscape-scale monitoring but often lacks resolution below dense canopies, limiting its ability to capture understory and lower trunk details^40^. Typical ALS campaigns, often acquired at moderate point densities (e.g., around 8–12 points/m²) and sometimes not fully temporally aligned with ground campaigns, may therefore offer limited capacity to resolve fine-scale canopy occlusions. Nonetheless, ALS provides critical context at the landscape scale and enables assessment of tree-level metrics across broader forest areas. Together, TLS and ALS offer complementary perspectives: TLS delivers highly detailed tree-scale structural information, while ALS extends these insights to larger spatial domains.

Despite these advancements, the development and validation of generalised tree structure models remain constrained by the limited availability of publicly accessible, high-quality published datasets containing labelled individual trees. Such datasets are vital for standardising model comparisons, evaluating algorithm performance, and supporting the training of machine learning models. Table 1 lists several such published datasets from different regions, showcasing a diversity of scanning platforms and forest types. Open-access datasets support collaborative research, facilitate replication, and accelerate advances in forest structure modelling.

**Table 1:** Published datasets of individual trees scanned by LiDAR across various regions, often used as references for algorithm evaluation and comparative studies.

Reference	Location	Number of trees	Platform
Weiser *et al*.^43^	Germany	1491	TLS, ULS and ALS
Owen *et al*.^66^	Spain	1441	TLS
Hollaus & Chen^67^	Austria	1328	TLS, MLS, ULS and ALS
Calders *et al*.^49^	United Kingdom	876	TLS
Krishna Moorthy *et al*.^68^	Panama and France	182	TLS
Disney *et al*.^69^	United Kingdom	92	TLS
Wan *et al*.^70^	China	73	TLS
Sanne *et al*.^71^	Belgium	69	TLS
Demol *et al*.^72^	Belgium	66	TLS
Calders *et al*.^73^	Australia	63	TLS
Momo Takoudjou *et al*.^74^	Cameroon	61	TLS
Hackenberg *et al*.^62^	Germany and China	36	TLS
Gonzales de Tanago Menaca *et al*.^75^	Guyana, Indonesia and Peru	29	TLS

TLS: Terrestrial Laser Scanning, MLS: Mobile Laser Scanning. ULS: Unmanned Aerial Vehicle (UAV) Laser Scanning, ALS: Aerial Laser Scanning.

While these datasets have significantly contributed to methodological development, they are predominantly from temperate ecosystems in Europe, the Americas, and Australia. There remains a distinct lack of similar datasets from tropical and subtropical regions, particularly from biodiversity-rich countries like India. Indian forests encompass a wide range of ecological zones, from tropical rainforests to dry deciduous and alpine forests, with diverse species compositions and highly variable structural characteristics. This ecological diversity presents both challenges and opportunities for advancing forest modelling techniques.

To address this geographic and data gap, we present a high-resolution LiDAR dataset collected from tropical and subtropical forests in northern India. The dataset includes detailed TLS-derived point clouds of 674 individual trees from 12 forest plots, representing 24 different species. For all plots, complementary ALS data are also provided, enabling multi-scale and multi-platform analyses of forest structure. Each tree is associated with rich metadata, including species identification, DBH and tree height.

This dataset offers an opportunity to benchmark and validate structural modelling approaches, especially in morphologically complex and underrepresented forest types, and serves as a valuable resource for remote sensing, forestry, and ecological communities. By contributing this open-access dataset, we aim to promote reproducible research, support model development and transferability, and advance the use of TLS and ALS in forest monitoring across data-scarce regions.

## Methods

This section details the comprehensive workflow for the acquisition, processing, and integration of Terrestrial Laser Scanning (TLS), Airborne Laser Scanning (ALS), and field data to generate a dataset of individual trees in the Shivalik Range, India. The methodology is structured into two parts:

- Part I: Dataset Generation — This part describes the data acquisition, preprocessing, co-registration, and integration steps used to generate labelled and segmented tree point clouds.
- Part II: Applications and Analyses — This part demonstrates the utility of the dataset in practical applications such as wood-leaf classification and structural metric derivation using established models and algorithms.

### Study area

**Fig. 1:**
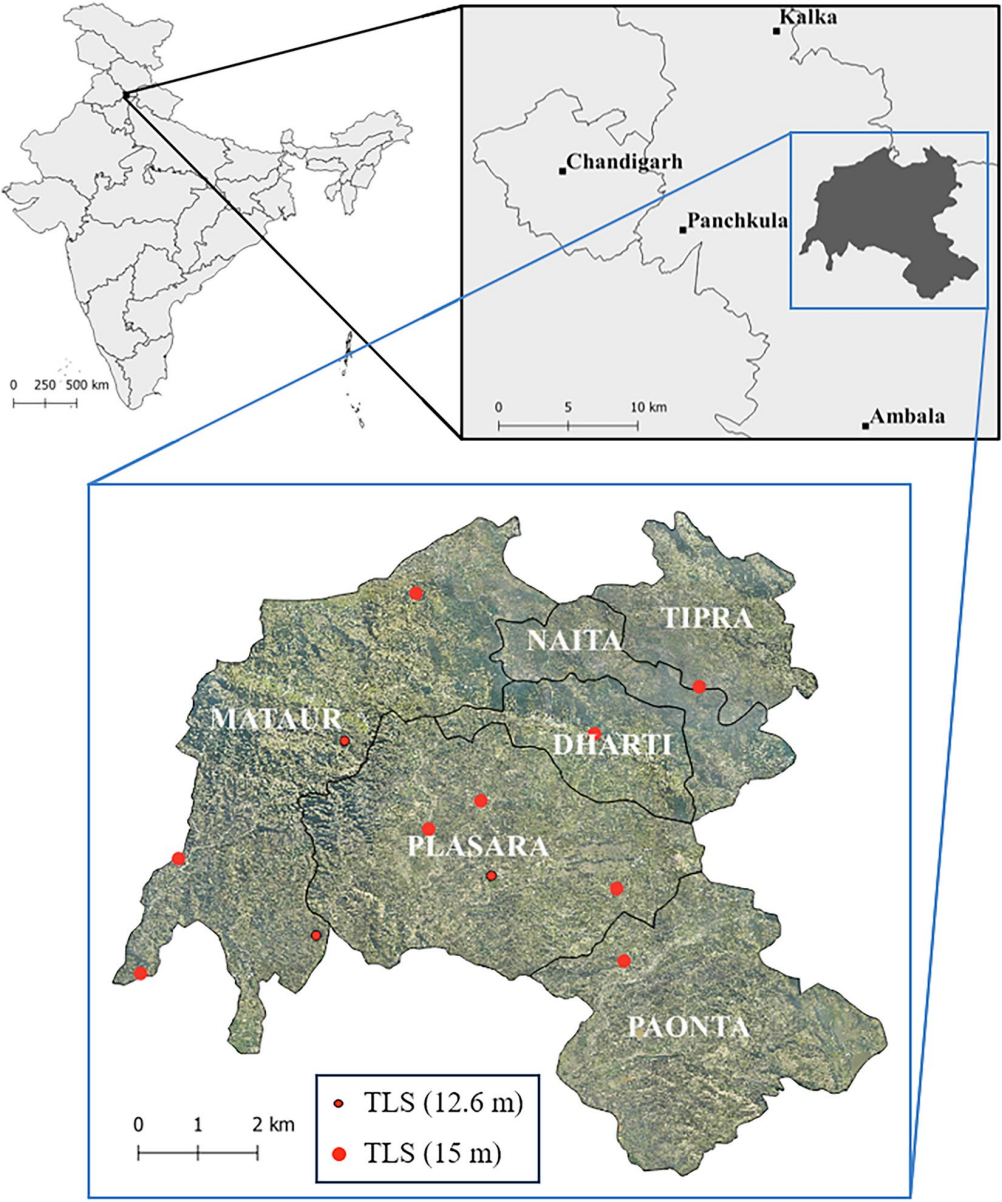
Location of the study area within the Shivalik Range of the Himalayan belt. The map highlights the districts managed by the Haryana Forest Department, with the specific forest plots selected for TLS indicated in red. The inset marked in blue shows the Bhoj clusters covered by ALS data. The TLS plots were circular, with radii of 12.6 m and 15 m, corresponding to plot areas of approximately 500 m² and 707 m², respectively.

The study was conducted in the hilly terrain of the Shivalik Range, part of the outer Himalayan belt, within the forest regions managed by the Haryana Forest Department in the northern circle districts of India (Fig. 1). Administratively, the area is divided into six clusters, locally known as Bhoj (village clusters or groups with shared administration). For TLS data collection, 12 forest plots were randomly selected across the study area.

Plot sizes were determined based on local terrain conditions and forest density, with circular plots having radii of either 15 meters (706.8 m^2^) or 12.6 meters (500 m^2^). These plots spanned a variety of topographies, including nearly flat areas as well as moderately to steeply sloped terrains. The data from 12 plots includes annotated point clouds of 674 individual trees, representing 24 species.

### TLS data acquisition and pre-processing

#### Data acquisition using TLS

TLS data were collected over 21 days between November and December 2023. Most trees were scanned under leaf-on conditions, although a few trees exhibited leaf-off characteristics due to natural seasonal shedding. A RIEGL VZ-2000i TLS scanner was employed, operating at a wavelength of 1550 nm, with a range measurement accuracy of 5 mm and a beam divergence of 0.27 mrad, resulting in a beam diameter increase of 27 mm per 100 m^41^.

Scans were performed with a pulse repetition frequency of 1200 kHz and an angular resolution of 40–50 mdeg. At a 100-meter range, this resulted in point spacings of approximately 70 mm for 40 mdeg resolution and 87 mm for 50 mdeg in a single scan. The scanner features a 360° horizontal and 100° vertical field of view (−40° to +60° from the horizontal plane).

Multiple TLS scans were taken from different positions, including slightly tilted scans, to capture the trees’ complete horizontal and vertical extent. In these cases, the scanner was manually tilted upward by adapting the tripod without using a tilt mount, only enough to improve crown coverage. Extreme orientations such as 90° tilts were not used, nor were they required. The scanner performed automatic onboard registration using a mounted Global Navigation Satellite System (GNSS) on top of the scanner, a built-in Inertial Measurement Unit (IMU), and a compass to align scan positions roughly. Point clouds were voxelized and merged automatically, followed by fine alignment using plane-patch matching. While reflective targets were not required for registration, they were strategically placed (11–35 per plot) and used as tie points for georeferencing. These were distributed to ensure optimal geometry for accurate transformation.

**Fig. 2:**
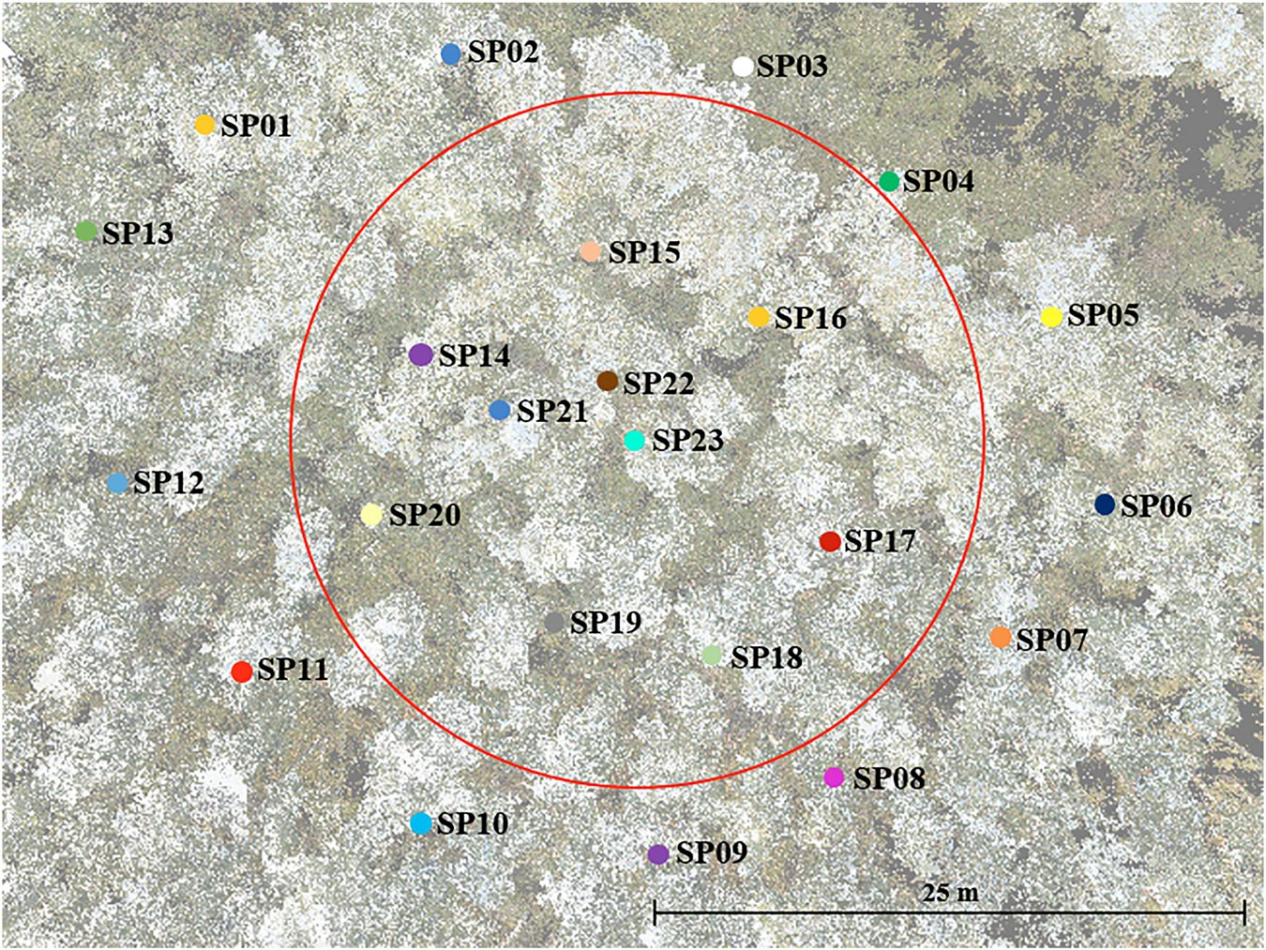
Top view of the Terrestrial Laser Scanning setup for one of the plots. The plot boundary is marked by a red circle, with individual scan positions (SP) distributed across the plot, starting from the outer boundary and progressing inward, culminating with the central scan (SP23). The background is a top-view snapshot of the plot’s point cloud as visualized in RiSCAN PRO, where the semi-transparent colours represent tree canopies and ground structures. This example is from a relatively uniform plot dominated by Eucalyptus tereticornis on nearly flat terrain.

Each plot had between 22 and 29 scans, depending on its size and complexity. The scanning pattern followed a concentric circular strategy, starting from the outside and proceeding inward, with a final scan at the plot centre, as illustrated in Fig. 2.

Some scans that failed onboard registration were manually registered in RiSCAN PRO^42^, followed by Multi Station Adjustment (MSA) using the Iterative Closest Point (ICP) algorithm based on planar features. Additionally, a Nikon D810 camera mounted atop the scanner captured 7 panoramic photos per scan to colourise the point cloud. Further, to improve the quality of the point cloud, filtering was applied to remove points with high pulse shape deviation, using a threshold value of 50.

#### Georeferencing the TLS Point cloud

Due to dense forest canopy blocking satellite signals, the TLS scanner’s onboard GNSS was insufficient for accurate positioning. Therefore, external GNSS receivers were used to obtain precise global coordinates. For each plot, three Trimble R10 GNSS receivers were placed outside the plot in areas with clear sky visibility, e.g., along roads or in adjacent agricultural fields. Observations were recorded for a minimum of two hours to ensure positional accuracy.

**Fig. 3:**
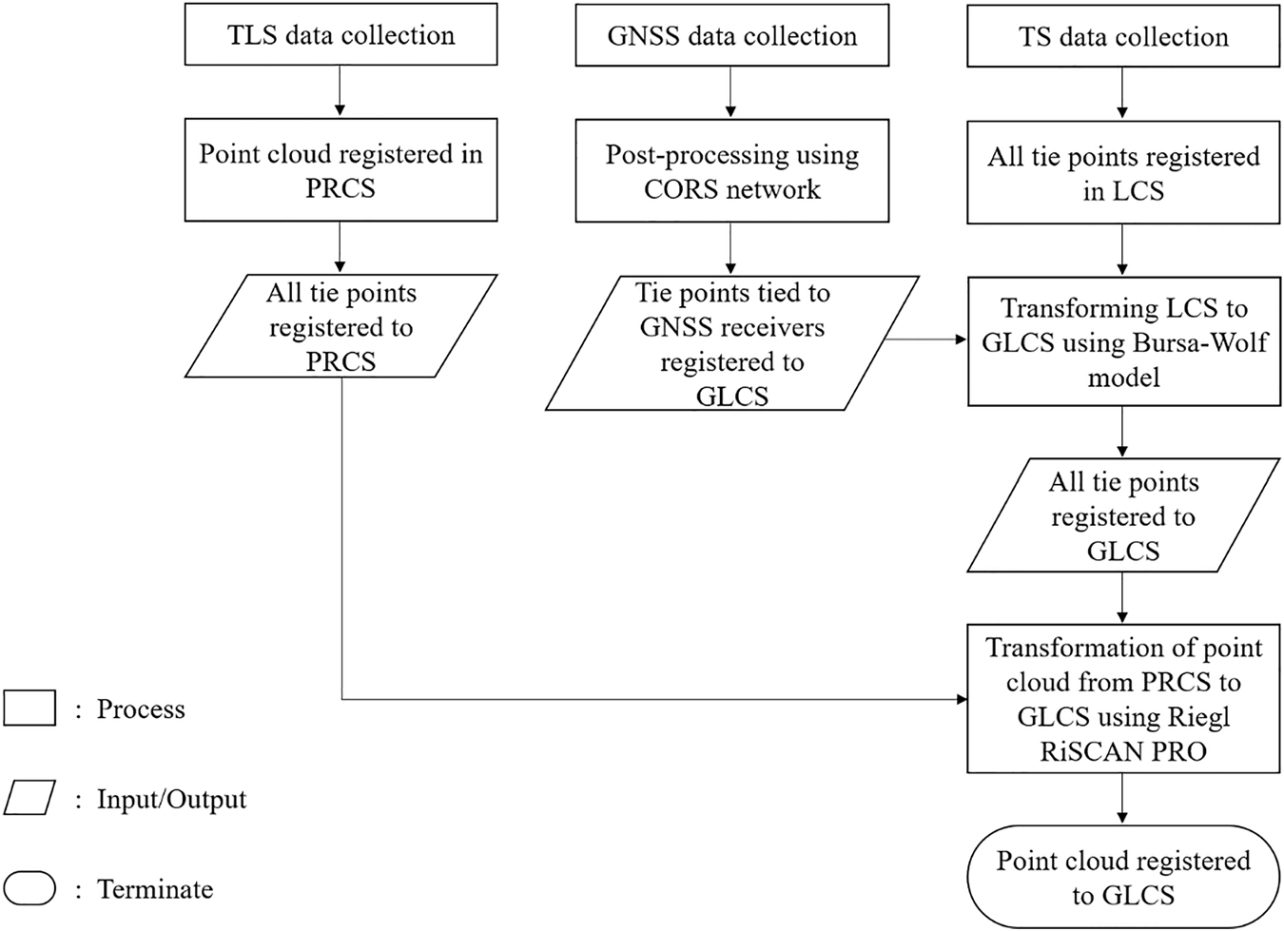
Flowchart illustrating the georeferencing methodology for TLS point cloud data, incorporating GNSS observations, Total Station measurements, and the Bursa-Wolf transformation model to convert local coordinates into global coordinates for accurate point cloud registration. PRCS: Projected Coordinate System, CORS: Continuously Operating Reference Station, GLCS: Global Coordinate System, LCS: Locally defined Coordinate System.

**Fig. 4:**
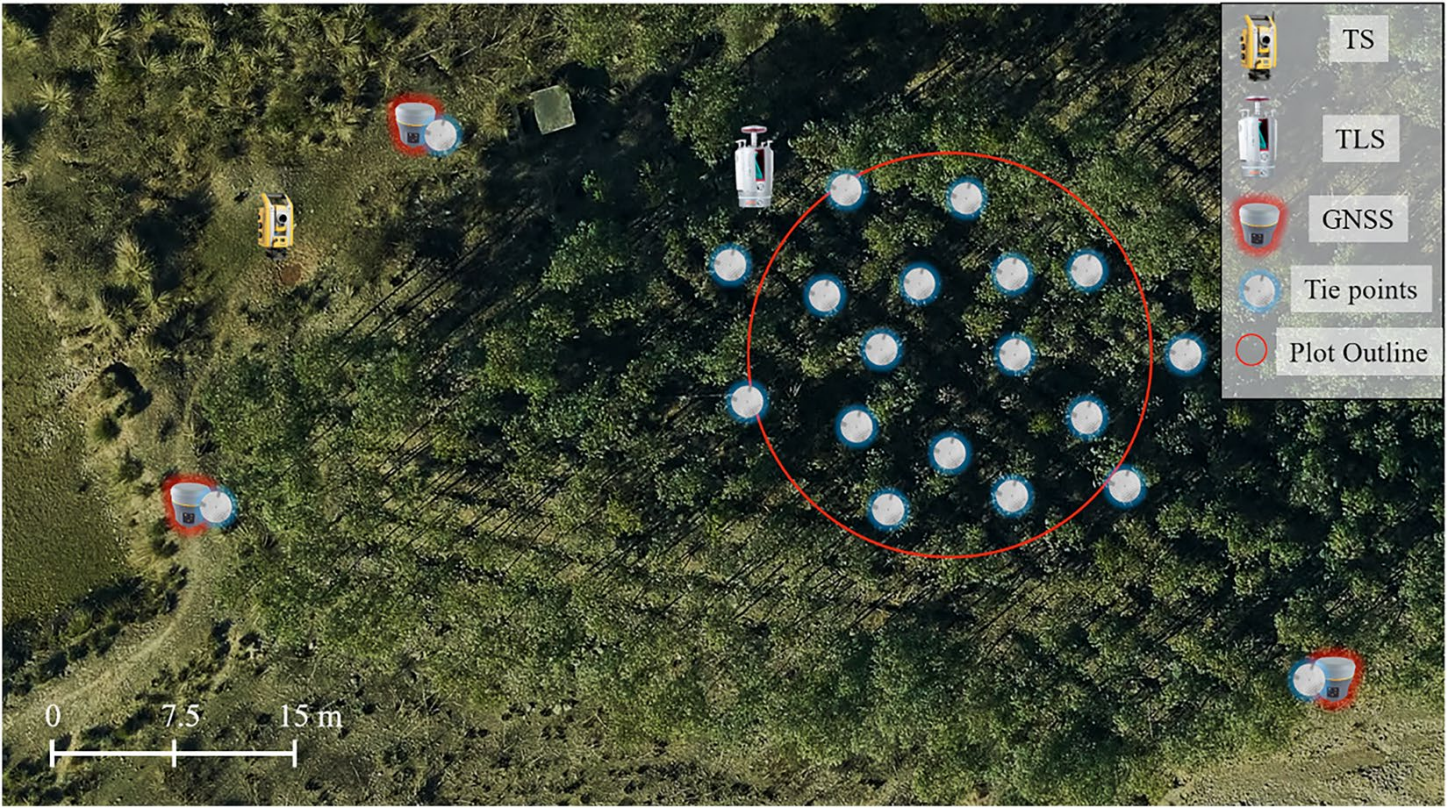
Schematic of the setup of GNSS receivers, Total Station (TS), and Terrestrial Laser Scanner (TLS) on one of the plots (Plot03) for georeferencing. While the GNSS receivers were kept stationary during data acquisition, the TS and TLS devices were repositioned multiple times across the plot to capture tie points and tree data from various locations.

A Trimble S5 Total Station (TS) was then used to transfer coordinates from these GNSS positions to tie points within the plot. Independent readings were taken from the TLS, GNSS, and TS, and post-processing was performed to convert local coordinates to global coordinates. This workflow is outlined in Fig. 3, with a typical setup illustrated in Fig. 4.

**Fig. 5:**
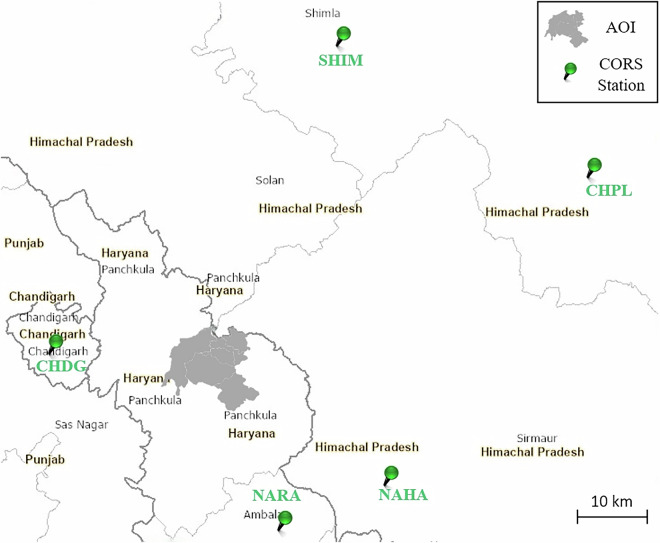
CORS network stations used for post-processing GNSS observations in the study area, including stations at Chandigarh (CHDG), Narayangarh (NARA), Nahan (NAHA), Chaupal (CHPL), and Shimla (SHIM), ensuring precise positional accuracy for georeferencing TLS point clouds. Yellow-shaded labels indicate state/UT names, while non-shaded labels indicate city names.

GNSS data were post-processed using the Survey of India’s (SOIs) CORS network, which included stations at Chandigarh (CHDG), Narayangarh (NARA), Nahan (NAHA), Chaupal (CHPL), and Shimla (SHIM), as shown in Fig. 5. Real-time correction was unavailable due to poor connectivity, so post-processed corrections were applied.

After processing the GNSS data using the CORS network, we obtained the global coordinates for GNSS receivers and the associated tie points. The TS was used to collect the local coordinates of all tie points, including those tied to GNSS receivers and trees within the plot. These local coordinates were then transformed into global coordinates using the tie points linked to GNSS receivers.

The transformation process employed the Bursa-Wolf model, which requires seven unknown parameters (three translations, three rotations, and one scale factor). For each plot, observations from the three GNSS receivers in both local (LCS) and global (GLCS) coordinate systems provided nine observations to solve these seven parameters, leading to the over-determined system and hence a least-squares solution was applied. The transformation equation is as follows:

1 $${[XYZ]}^{T}=\lambda {{R}}_{\kappa \phi \omega }{[xyz]}^{T}+{[{T}_{X}{T}_{Y}{T}_{Z}]}^{T}$$

Where *X, Y, Z* are global coordinates, *x, y, z* are local coordinates, *λ* is the scale factor, and ${T}_{X},\,{T}_{Y},\,{T}_{Z}$ represent the translations along the *X, Y* and *Z* axes. The matrix ${R}_{\kappa \phi \omega }$ is a combined rotation matrix incorporating three 2D rotation matrices for rotations about the $X,{Y}$ and $Z$ axes.

By applying this model, we transformed the local TS coordinate system into the global coordinate system. As a result, the tie points collected by the TS were also converted into global coordinates, allowing them to be used for georeferencing the TLS point cloud.

The same model was applied in the Riegl RiSCAN PRO software to transform the TLS point cloud from the PRCS to the GLCS. The tie points, which served as Ground Control Points (GCPs) distributed throughout the plot, were fine-scanned during TLS data collection, resulting in tie points in PRCS. The corresponding GLCS coordinates of these points, calculated using TS and GNSS as described above, were used to georeference the TLS point cloud.

### Data acquisition using ALS

**Fig. 6:**
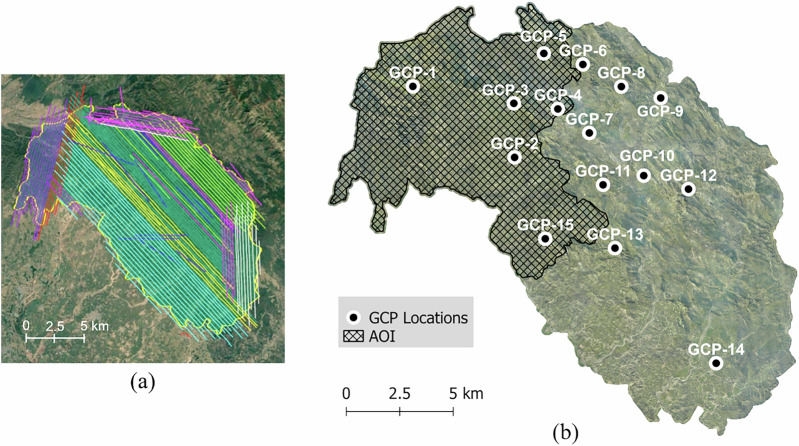
Coverage of Aerial Laser Scanning and orthophoto survey over the 250 km² survey area. (**a**) Flight lines of the aerial survey. (**b**) Locations of the 15 GCPs distributed across the survey area. The hatched region highlights the 100 km² area of interest where TLS sample plots were also established (see Fig. 1 for plot locations within this AOI).

ALS data were acquired by the Haryana Forest Department, India, through Geokno Pvt. Ltd. over an area of approximately 250 km^2^, as shown in Fig. 6. The data was collected in February 2022 using a Riegl LMS-Q780 LiDAR scanner, integrated with a 100 MP Phase-1 IXU R 1000 camera, mounted on a VT-GVF Eurocopter AS350-B3 Ecureuil helicopter. The acquisition was conducted over 118 flight strips, and the datasets were georeferenced using 15 GCPs. The GCPs were distributed across all Bhoj clusters to ensure uniform coverage of the area of interest. Each GCP was marked in black and white with an outer circle of 1 m radius and an inner circle of 35 cm, designed for clear visibility in aerial imagery and LiDAR data. A static GNSS base station was established near GCP-01, connected to the SOI reference point at Chandigarh, and supported by a backup station. GNSS data were processed using the Post-Processing Kinematic (PPK) method, achieving centimetre-level positional accuracy. The final point cloud was georeferenced to the UTM zone 43 (EPSG:32643) coordinate system, with orthometric heights derived using the EGM2008 geoid model. The average point cloud density was approximately 8 points/m² in non-overlapping areas of flight lines.

Alongside ALS acquisition, aerial imagery captured with the Phase-1 camera was processed to generate high-resolution true orthophotos at 10 cm ground sampling distance (GSD). Aerial triangulation (AT) was performed using exterior orientation parameters, camera calibration data, and the 15 GCPs to refine alignment. LiDAR-derived Digital Terrain Models (DTMs) and Digital Surface Models (DSMs) were then employed for ortho-rectification, ensuring removal of terrain and building distortions. Individual orthophoto tiles (1 × 1 km) were mosaicked into a seamless product using colour and brightness balancing.

To facilitate integration with TLS data, circular clips were extracted around each of the 12 TLS plots, with an additional 2-meter buffer added to ensure the inclusion of boundary trees. Individual tree point clouds were then manually segmented from the clipped ALS data. Although ALS data were collected for the entire 250 km² area, the dataset presented here includes only those ALS point clouds corresponding to trees co-registered with TLS-derived trees in the 12 selected plots. Those interested in accessing the broader ALS dataset for regions beyond the TLS plots may contact the authors or the Haryana Forest Department.

### Alignment of ALS point cloud to TLS point cloud

TLS point clouds were processed and projected onto the UTM zone 43 (EPSG:32643) coordinate system to match the ALS data, which had already been georeferenced to the same coordinate system during acquisition. The EGM2008 model was used for converting elevation values to orthometric heights. Despite using a common coordinate system, slight misalignments were observed between the datasets. These discrepancies likely arose due to variations in GNSS accuracy, differences in georeferencing workflows, and inherent positional errors in ALS and TLS data.

The ICP algorithm was applied to correct these misalignments. As a prerequisite, identifiable man-made structures in both point clouds were extracted to serve as reference features for computing transformation parameters. Due to the forested nature of the site, only a limited number of such structures were available, particularly within the TLS plots. Nevertheless, the computed transformation parameters were applied to effectively align the ALS point cloud with the TLS data.

We chose TLS as the reference frame for alignment because it was georeferenced with higher accuracy. Each TLS plot was positioned using static GNSS observations, post-processed against the CORS network, and further refined using a total station, ensuring centimetre-level precision. In contrast, ALS data, although georeferenced with GCPs and aerial base station corrections, remains more susceptible to systematic errors from flight dynamics and platform geometry. Thus, ALS was aligned to TLS to ensure that the finer-resolution TLS data served as the more stable reference for co-registration.

### Individual tree segmentation

**Fig. 7:**
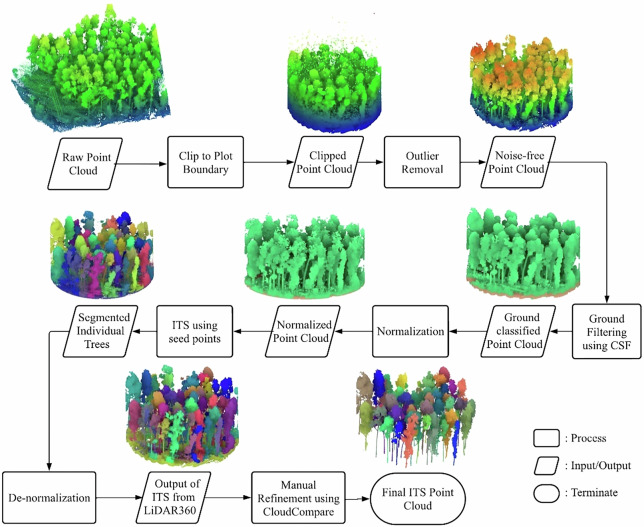
Workflow for Individual Tree Segmentation from TLS point cloud, illustrating the steps from initial clipping and outlier removal to automatic segmentation and subsequent manual refinement. All subfigures depict the same representative plot section. Minor adjustments in viewing angle were intentionally applied between panels to better visualise the results of each processing step, particularly for ground classification and normalised point cloud visualisation, while preserving the same spatial subset throughout.

Segmentation of 674 individual trees from the TLS point cloud was performed using a combination of automatic extraction and manual refinement, an approach commonly employed in previous studies to improve segmentation accuracy in structurally complex forests^43,44^. The process starts with automatic segmentation using LiDAR360^45^, followed by manual corrections to address inaccuracies caused by the understory and overlapping crowns. The complete workflow is illustrated in Fig. 7.

The process began with clipping the TLS point cloud to plot boundaries to retain only the relevant tree data, followed by noise removal. Specifically, points with high pulse shape deviation were filtered out using a threshold value of 50, after which isolated random points floating above the canopy were manually removed. Ground points were identified using the Cloth Simulation Filter (CSF), and a DTM was generated using LiDAR360. The point cloud was then normalised using the DTM, as normalisation is essential for optimal segmentation performance in LiDAR360.

Seed points were manually selected by slicing the normalised cloud at 1.3 m above ground level, where tree trunks appear circular. Using these seed points, LiDAR360 segmented individual trees. The segmented point cloud was subsequently denormalised to convert normalised heights back to absolute values. However, this automatic process introduced several errors, such as merging adjacent trees, misclassifying non-tree points, and imprecise crown boundaries, especially in cases of crown overlap, dense understory, and occlusions. Errors also stemmed from incorrect seed point placement.

To mitigate these issues, manual refinement was performed using the interactive segmentation tool in CloudCompare^46^, which involved comparing each tree to its surrounding trees. Combining automatic segmentation with manual correction resulted in precisely segmenting individual trees from the TLS point cloud.

For the ALS point cloud, the same 674 trees were segmented manually using CloudCompare. The TLS-based segmentation was used as a reference, and trees were carefully extracted from the ALS point cloud post-alignment. This ensured accurate correspondence between ALS and TLS-derived tree segments for subsequent analyses.

### Field measurements

Field measurements were collected simultaneously with TLS data acquisition for all 342 trees in the study that had a DBH greater than 10 cm. DBH was measured at 1.3 m above the ground using a measuring tape. For leaning or non-vertical trees, measurements were taken 1.3 m along the trunk axis from the base.

In addition to DBH, species identification was recorded for these 342 trees. Photos of the bark, leaves, and full tree were also taken. Each tree was marked with a unique identifier painted directly on the trunk, which was clearly visible in the RGB-coloured TLS point cloud. This enabled an accurate association between field measurements and individual tree point clouds. Additionally, tie points were assigned to these trees, further aiding the alignment of field-labelled trees with their corresponding point clouds.

The primary species identified were *Eucalyptus tereticornis*, *Acacia catechu*, *Holoptelea integrifolia*, *Dalbergia sissoo* and *Tectona grandis*. A complete list of species, along with their counts, height ranges, and total volumes, is provided in Table S1 of the supplementary file.

### Wood-leaf separation on TLS data

To provide an indirect quality assessment of the TLS dataset, we applied several established wood–leaf separation algorithms to the individual tree point clouds. Manual wood–leaf separation was first performed using the interactive segmentation tool in CloudCompare, and these manually classified point clouds served as the ground truth for evaluating the automated algorithms. While manual interactive segmentation offers a reliable reference, it is not entirely free from uncertainty. Labelling errors may occur in areas of dense foliage, shadowed stems, or occluded branches, and the process is inherently time-consuming and subject to operator bias. Nonetheless, such expert-guided segmentation remains the most practical means of generating consistent ground-truth data for validating leaf–wood classification algorithms and, by extension, assessing dataset integrity.

The rationale for using wood–leaf classification performance as a proxy for dataset quality is that these algorithms rely heavily on the geometric consistency of the point cloud. High-quality TLS datasets, characterised by dense, evenly distributed points, minimal noise, and accurate co-registration, enable reliable computation of geometric features such as curvature, planarity, and verticality that underpin wood–leaf discrimination. Consequently, the classification accuracy achieved by well-established algorithms serves as an indicator of how faithfully the dataset captures fine-scale tree structure. Lower accuracies, by contrast, may suggest issues such as occlusion, uneven sampling, or registration artefacts that degrade geometric feature computation. Our aim here was not to introduce new methods or provide state-of-the-art benchmarking, but rather to demonstrate that the dataset behaves consistently with results previously reported in Ali *et al*.^15^ on widely used open-source TLS benchmarks.

Specifically, we implemented the same four algorithms evaluated in Ali *et al*.^15^, LeWoS, TLSeparation, CANUPO, and Random Forest (RF), and applied them to our dataset using identical parameter settings. For LeWoS, the default verticality threshold of 0.125 was used. TLSeparation required configuration of parameters like voxel sizes, k-nearest neighbours (knn), and retrace steps, for which default values were retained. CANUPO was applied with a multi-scale descriptor range from 0.1 to 1.0, in 0.1 increments. The RF model was configured using 12 ad-hoc features described in Fig. 3 of Ali *et al*.^15^, including anisotropy, PCA2, surface variation, sphericity, verticality, and relative height, computed at multiple neighbourhood radii. The final dataset includes both the unfiltered (unclassified) point clouds and the corresponding wood-only (leaf-filtered) version derived from the manual wood-leaf separation.

### Metrics derived from point clouds

Metrics such as tree height, DBH, and volume were derived from both ALS and TLS point clouds. Tree height was computed as the difference between the maximum and minimum Z-values within each segmented tree, a standard method applied in many LiDAR-based forest studies^38,47^. Each tree was carefully delineated and manually refined through visual inspection to ensure that both the apex and the true base of the tree were included, effectively capturing the actual ground level beneath each tree. This manual validation ensured that minimum Z-values corresponded closely to the true ground/base of each tree, even in cases where ALS ground returns might be sparse. Thus, max–min Z provided reliable tree height estimates consistent with the underlying field structure.

Tree height was estimated from both datasets, while DBH and volume were computed only from the TLS point clouds due to their higher spatial resolution and reduced occlusion. The TreeQSM model^48^ was used for DBH and volume estimation, which has been successfully employed in several studies for reconstructing tree architecture and volume estimation^7,9,12,13,35,48–50^. TreeQSM fits cylindrical primitives to point clouds to reconstruct tree architecture. Parameters including PatchDiam1, PatchDiam2Min, and PatchDiam2Max were tuned to local forest conditions. One-fourth of the trees (up to 10 trees) were used for parameter optimisation, leveraging TreeQSM’s built-in optimisation capability. Following the recommendations of Ali *et al*.^12^, the number of optimisation values was set to 2 for PatchDiam1, 4 for PatchDiam2Min, and 3 for PatchDiam2Max, balancing accuracy and processing time.

For trees with buttressed bases, a hybrid approach combining Poisson Surface Reconstruction (PSR) and TreeQSM was applied, as described in Ali *et al*.^12^, to achieve more accurate volume estimation. To minimise variability from TreeQSM’s stochastic outputs, each tree was processed ten times using the same parameter set, and average DBH and volume values were recorded.

## Data Record

The dataset collected in this study is made publicly available through the Zenodo repository^51^, and the LiDAR point cloud repository, LiDAVerse (www.LiDAVerse.com). It provides a comprehensive set of forest structural data from Indian tropical and subtropical ecosystems, curated to support a wide range of ecological, remote sensing, and modelling studies. The data are structured into the following components:

- Terrestrial Laser Scanning (TLS) Data
  Individual tree-level point clouds for 674 trees, captured using high-resolution TLS, and processed for direct use. Each point cloud retains standard attributes including intensity, return number, number of returns, and GPS time.
  Manually leaf-filtered TLS point clouds are provided as separate files. These include only wood points, curated specifically for tree structure modelling and volume estimation. Leaf-filtering was performed only for trees with DBH greater than 10 cm, as smaller trees have limited volumetric significance.
  Full plot-level TLS point clouds (including understory vegetation, ground points, and unsegmented trees), as well as the raw unprocessed TLS scans, are available upon request from the authors.A small number of leaf-filtered TLS files contain localised zero intensity values, which are not present in the corresponding unfiltered datasets. This minor attribute loss likely occurred during the manual segmentation and export process, where intensity values were partially reset for some points. The original unfiltered TLS files retain complete and valid intensity data.
- Airborne Laser Scanning (ALS) Data
  Individual tree-level ALS point clouds co-registered with TLS data for the same 674 trees.
  ALS data for the broader study area (~250 km²) are available upon request from the authors or the Haryana Forest Department.
- Ground Observations and Species Data
  Field measurements for 342 trees with DBH > 10 cm, including DBH and species identification.
  RGB photographs of bark, leaves, and full-tree profiles to support species classification tasks. As homogeneous plots (e.g., Plot01, Plot03) required only representative samples, not all trees have associated photographs. The photos are stored in a separate folder.
- Aerial Orthophoto
  High-resolution orthophotos covering the study plots, useful for crown delineation and landscape context.
- Plot Metadata and Tree Metrics File
  A plot metadata file (Plot_Details_PlotID.txt) is provided for each TLS plot, containing detailed plot-level information such as the plot ID, approximate coordinates (WGS84), data acquisition details (type of data collected, date, and personnel), and a brief description of the plot environment, including vegetation type, terrain, and local conditions influencing scanning.
  The metadata file also records the key TLS survey parameters, including scanner model, angular resolution, pulse frequency, number of scan positions, RGB imaging, and plot radius.
  A single consolidated tree metrics file (Tree_Metrics.csv) contains tree-level attributes for all plots combined. Each record includes PlotID, TreeID, Species, Height_TLS, Height_ALS, DBH_field, DBH_TLS, and Volume, enabling cross-comparison between field, TLS-, and ALS-derived structural measurements.
  Together, these files ensure complete traceability between plot-level metadata and tree-level structural parameters, providing a comprehensive link between field observations and LiDAR-derived data products.

**Table 2:** Dataset organisation and file naming convention.

Component	Description	Example
Folder Structure	Organised by plot and data type	/Plot01/, /Photos/, etc.
SensorType	Type of LiDAR sensor used: Terrestrial (TLS) or Airborne (ALS)	TLS or ALS
SpeciesName	Initials of species name	EucTer for *Eucalyptus tereticornis*
PlotID	Unique ID for each plot	Plot01, Plot02, etc.
TreeID	Unique ID for each tree; understory trees prefixed with “U”, outer boundary trees with “O”	003, U02, O01
Date	Date of acquisition in YYYYMMDD format	20240422
LeafFiltering	Indicates if the point cloud is leaf-filtered or unfiltered	Leaf-Filtered, UnFiltered
PhotoType	Type of photo captured: Bark (close-up of trunk), Leaf (close-up of leaves), or FullTree (whole tree profile)	Bark, Leaf, FullTree
Example Filename	Complete structured filename	TLS_EucTer_Plot03_007_20240315_Leaf-Filtered.laz

The files are organised as described in Table 2 in a hierarchical folder structure as follows:
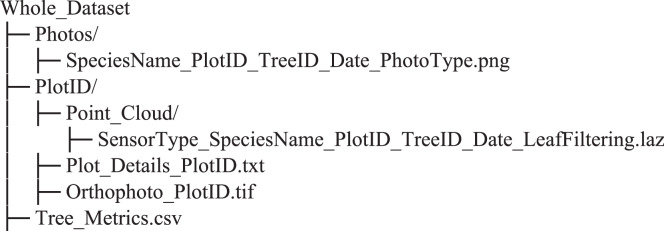


## Technical Validation

### Positional accuracy and georeferencing accuracy of TLS point clouds

Independent GNSS observations were recorded at three reference points per plot, placed in clear-sky areas outside the dense forest canopy to minimise signal obstruction. Post-processing using the CORS network, comprising stations at Chandigarh (CHDG), Narayangarh (NARA), Nahan (NAHA), Chaupal (CHPL), and Shimla (SHIM), significantly enhanced the positional accuracy. The standard deviation of positional precision ranged from 4.7 cm to 20.2 cm across plots, as presented in Table 3.

**Table 3:** Positional precision (standard deviation in cm) of GNSS observations from three reference points per plot after post-processing.

Plot ID	SD (GNSS1)	SD (GNSS2)	SD (GNSS3)
1	5.1	5.2	4.7
2	5.1	6.2	5.7
3	5	5.3	5.8
4	5.8	5.2	5.6
5	10.9	23.9	19.2
6	20.2	10.4	18.1
7	5.1	5.8	10.2
8	5.7	7	7.4
9	5.7	6.5	5.4
10	6.1	4.5	5.7
11	5.7	6	5.7
12	5.5	5.6	5.4

TS measurements were initially acquired in a local coordinate system and later transformed into the global coordinate system using the seven-parameter Bursa-Wolf model. GNSS observations from three control points per plot enabled estimating these parameters via a least-squares solution, resulting in centimetre-level transformation accuracy.

As illustrated in Fig. 3, georeferenced tie points were first established at GNSS receiver locations placed in open areas outside the plots. These global coordinates were then transferred to additional tie points inside the plot using a TS, which recorded their local coordinates relative to the GNSS reference points. The TLS point cloud, initially registered in a local PRCS, was then transformed into the GLCS using these tie points. This transformation, executed in RiSCAN PRO, involved estimating transformation parameters from matched local and global tie point coordinates. The software reported the standard deviation of transformation residuals for each plot, which ranged from 9.1 mm to 19.8 mm, as shown in Table 4.

**Table 4:** Number of Tie Points and Standard Deviation of Residuals (mm).

Plot ID	Number of Tie Points	SD of Residuals (mm)	Plot ID	Number of Tie Points	SD of Residuals (mm)
1	11	15.9	7	18	13.1
2	15	11.8	8	26	9.1
3	12	19.8	9	35	11.2
4	23	13.5	10	19	12.4
5	23	14.7	11	22	13.6
6	15	15.1	12	23	13.1

### Co-Registration of Individual TLS Scans

Multiple scan positions within each plot were initially aligned using coarse registration based on the TLS instrument’s onboard GNSS and IMU. This preliminary alignment was refined using an internal ICP algorithm. For high-precision alignment, the MSA algorithm in RiSCAN PRO was applied. The registration quality was evaluated through a two-stage process.

In the first stage, the quality of registration was assessed based on the standard deviation of residuals from the MSA performed in RiSCAN PRO for each plot, with values listed in Table 5. These values indicate centimetre-level accuracy, with the Mean Absolute Deviation (MAD) ranging from 12 mm to 23 mm.

**Table 5:** Results of the two-stage registration accuracy assessment for TLS scans.

Plot ID	First Stage Assessment		Second Stage Assessment	
	Number of Scans	MAD (mm)	Horizontal Deviation (mm)	Vertical Deviation (mm)
1	23	17	2.65	4.71
2	26	20	1.38	8.62
3	23	23	3.15	6.07
4	25	20	4.76	3.0
5	29	17	1.51	7.63
6	22	15	0.63	5.39
7	24	15	1.72	3.73
8	28	15	1.14	4.86
9	26	16	1.21	3.61
10	22	12	2.14	2.21
11	26	21	2.43	3.34
12	20	13	1.92	4.86

The first-stage evaluation represents the residuals from the Multi Station Adjustment performed in RiSCAN PRO, while the second-stage evaluation was conducted at the individual-tree level. In the second-stage assessment, horizontal deviation was computed from a circular stem slice projected onto the XY plane, and vertical deviation was derived from a second-order branch section projected onto the vertical plane (XZ or YZ).

In the second stage, a manual assessment of the registration accuracy of the point clouds from different scan positions was conducted to evaluate both horizontal and vertical registration accuracy. For horizontal accuracy, trunk points within a 0.2 m to 0.4 m section from the bottom of a centrally located tree in each plot were segmented and projected onto the XY plane, as shown in Figure S1 of the supplementary file. This process was repeated for all scans individually to analyse segmented points across different scan positions. Two point clouds from different stations were loaded and projected onto the XY plane, with a quadratic curve applied to fit the data. These curves modelled the structure of the point cloud, using the vertex of the parabola to represent the curve centre. Deviations between corresponding points were computed using radial directions, and the mean deviation quantified alignment accuracy.

For vertical registration accuracy, a second-order branch from the same tree was selected and projected onto the vertical plane (XZ or YZ plane), as shown in Figure S2 of the supplementary file. A linear fit was applied to a near-linear section of the branch in point clouds from different stations, and the perpendicular distance between the fitted lines was measured to assess vertical alignment. Mean deviations ranged from 0.63 mm to 4.76 mm for horizontal accuracy and 2.21 mm to 8.62 mm for vertical accuracy, as detailed in Table 5.

For Plot 4, the terrain consists of highly undulating terrace steps from historic farming practices. This caused some difficulty in perfectly co-registering all TLS scans, leading to a few small clusters slightly misaligned with the main point cloud. The higher horizontal deviation observed for Plot 4 in Table 5 reflects this effect. These clusters are visually well separated and are provided as-is for completeness; users can easily identify and, if necessary, remove them during analysis.

### Quality assessment of ALS data

To evaluate the absolute vertical accuracy of the ALS data, independent observations from three GNSS receivers per plot were used. A local ground mesh was generated at each GNSS base position, and the corresponding orthometric heights, obtained via post-processing as outlined earlie, served as reference values.

**Fig. 8:**
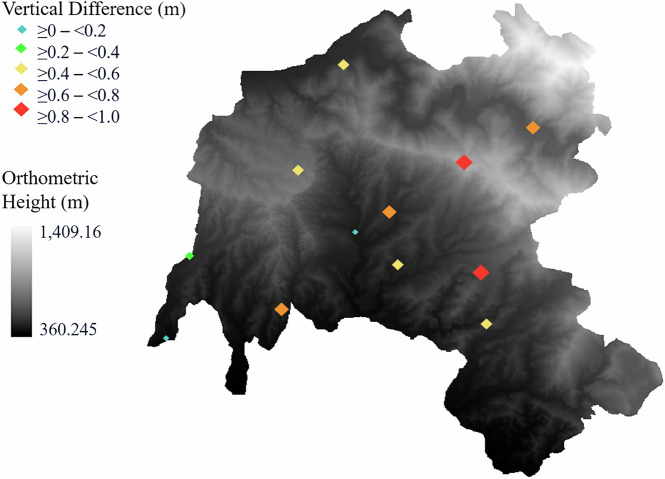
Comparison of ALS-derived ground heights and GNSS-derived orthometric heights at GNSS receiver locations across plots. The average height difference is displayed, overlaid on a Digital Terrain Model (DTM) with orthometric heights ranging from 360.24 m to 1409.16 m.

For each GNSS receiver, the height difference between the ALS-derived DTM and the GNSS-observed orthometric height was computed. This analysis provided a plot-wise assessment of ALS elevation accuracy and is visualised in Fig. 8. Although approximate, this analysis was intended to provide a rough check on the absolute vertical accuracy of the ALS data across the plots.

### Registration of ALS data to match TLS data

Since ALS and TLS datasets were acquired and georeferenced independently, slight spatial misalignments were observed between them. To achieve precise alignment, the ICP algorithm was employed, treating the ALS point cloud as the source (transforming) dataset and the TLS point cloud as the target (fixed) reference.

The ICP algorithm was iteratively executed five times, using stable man-made structures (e.g., building roofs) as alignment features. This procedure reduced the RMSE to 0.274 m, indicating substantial improvement in dataset alignment.

**Fig. 9:**
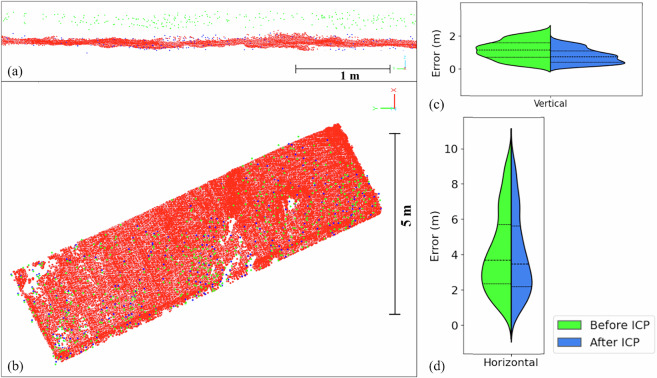
Assessment of ALS–TLS alignment before and after ICP transformation. Red points represent TLS data, green points represent ALS before ICP, and blue points represent ALS after ICP. (**a**) Vertical profile showing misalignment prior to correction. (**b**) Plan view illustrating residual XY discrepancies. (**c**) Violin plot of vertical errors, showing reduced spread and lower median error after ICP. (**d**) Violin plot of horizontal errors, indicating improved alignment following ICP. In the violin plots, dashed lines represent median error values, while the upper and lower dotted lines indicate the interquartile range (25^th^–75^th^ percentiles).

To further evaluate the alignment quality, an independent validation was performed using a point cloud section that was not included in the transformation process. Horizontal RMSE, vertical RMSE, and overall RMSE were computed as 0.15 m, 0.05 m, and 0.16 m, respectively. The effectiveness of ICP alignment is shown in Fig. 9, where violin plots illustrate the distribution of absolute horizontal and vertical misalignments before and after transformation. The results clearly demonstrate a reduction in both median and overall error spread after ICP, with vertical misalignments improving more substantially than horizontal ones.

### Tree metrics extracted from point clouds

Tree-level metrics derived from the point clouds included height, DBH, and volume from TLS, and tree height from ALS. Due to the absence of ground-truth data for tree height, direct validation was not possible. Furthermore, the ALS (February 2022) and TLS (November 2023) datasets were acquired nearly two years apart under different acquisition conditions (sensor type, viewing geometry, and point density). These factors, combined with natural tree growth, make it challenging to disentangle how much of the observed differences are due to true growth versus variations in data characteristics.

**Fig. 10:**
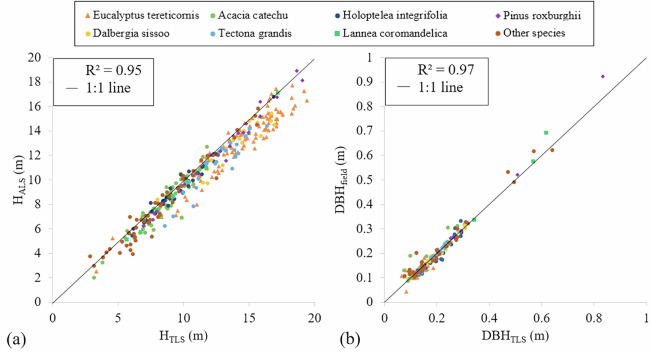
Correlation of (**a**) tree height (H) derived from TLS and ALS and (**b**) DBH derived from TLS and field measurements.

Field-measured DBH values were available for trees with DBH > 10 cm and served as a benchmark for TLS-derived DBH estimates. The analysis revealed strong correlations between the datasets (Fig. 10). TLS-derived tree heights were highly correlated with ALS-derived heights (R² = 0.95), with a mean deviation of 0.91 m. Similarly, TLS-derived DBH values correlated well with field measurements (R² = 0.97), with an RMSE of 1.9 cm, as illustrated in Fig. 10, but this difference reflects both potential growth and acquisition-related discrepancies.

**Fig. 11:**
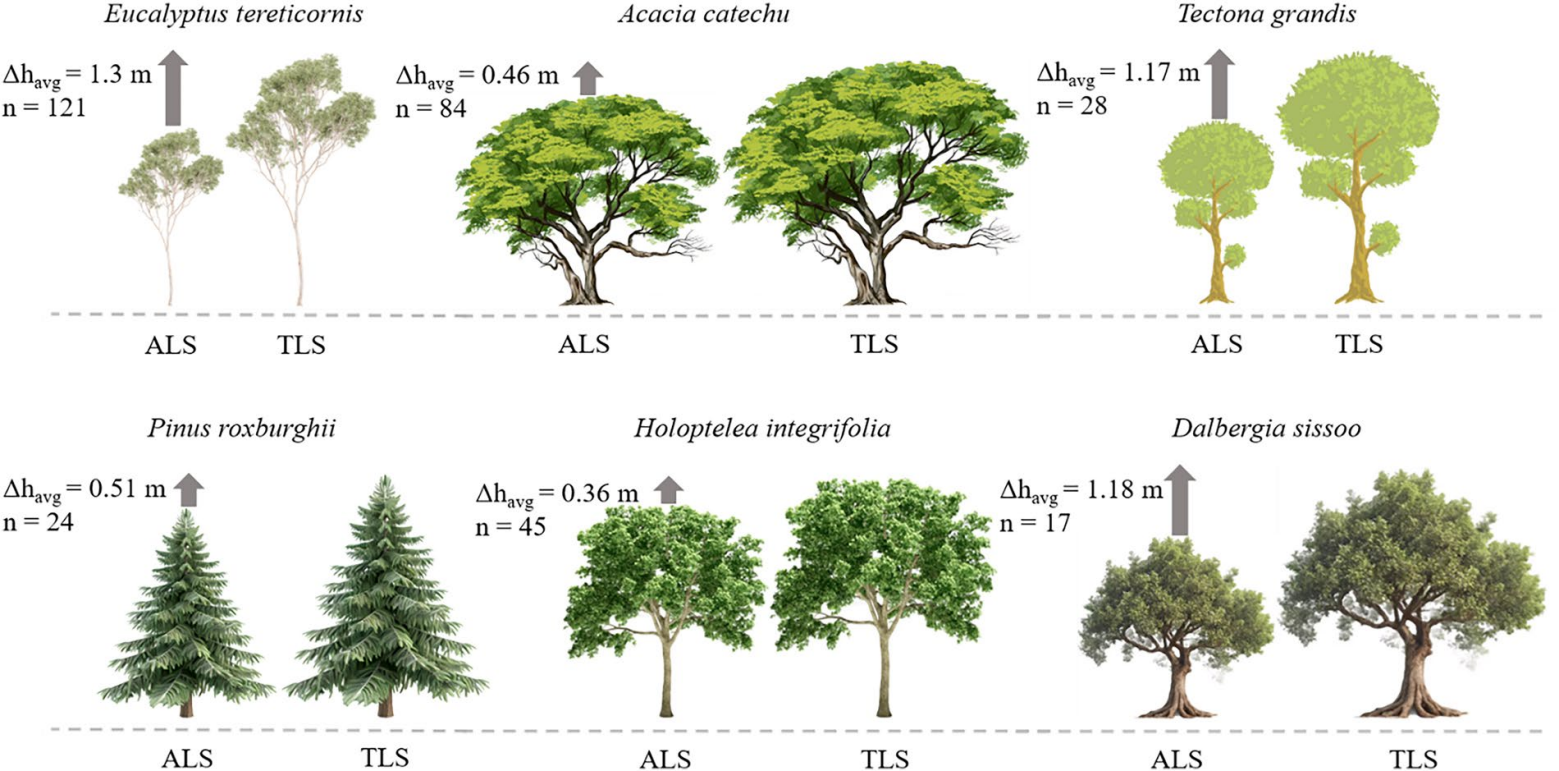
Mean tree height increment (Δh_avg_) estimated over a two-year period, based on ALS data acquired in February 2022 and TLS data acquired in November 2023. For each species, values represent the mean of ‘n’ trees. The figure displays only the six dominant species out of the 26 species present in the dataset.

Given these uncertainties, the observed differences in tree height between the two datasets were interpreted cautiously. To explore potential growth trends, an indicative height increment over the two-year period was calculated from ALS- and TLS-derived heights (Fig. 11). While these results suggest that species such as *Eucalyptus tereticornis*, *Dalbergia sissoo*, and *Tectona grandis* exhibited larger positive differences (mean ~1.3 m, 1.18 m, and 1.17 m, respectively), these values should not be interpreted as precise growth estimates.

In some cases, TLS-derived heights appeared overestimated relative to ALS-derived heights, especially for trees with broad crowns or when the ALS failed to capture the highest canopy returns due to coarser point spacing. Conversely, underestimation occurred when TLS could not fully capture the upper canopy or true apex because of occlusion by branches or neighbouring trees. These discrepancies reflect both differences in acquisition geometry and temporal offsets as well as the natural structural complexity of tropical forests.

Other species showed smaller differences (mean ~0.51 m for *Pinus roxburghii*, 0.46 m for *Acacia catechu*, 0.36 m for *Holoptelea integrifolia*, and 0.45 m for other species). Overall, the trend indicates that taller, fast-growing species exhibited larger positive deviations, consistent with both expected growth and sampling-geometry effects.

**Fig. 12:**
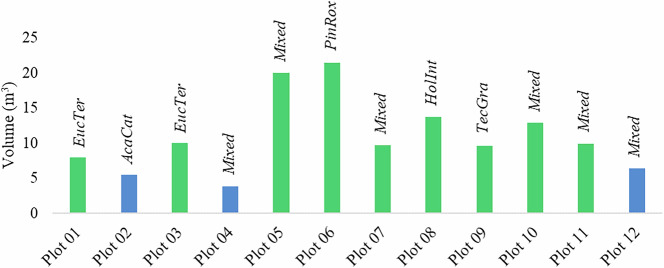
Plot-wise total tree volume derived from TLS data. Each bar represents the sum of individual tree volumes within a plot, with labels above indicating the dominant species. Blue bars denote plots with a 12.6 m radius, while the green bars correspond to plots with a 15 m radius. Species abbreviations follow those provided in Table S1 (Supplementary Material), which lists all 26 species and their corresponding abbreviations. Example abbreviations include *EucTer* (*Eucalyptus tereticornis*), *AcaCat* (*Acacia catechu*), *PinRox* (*Pinus roxburghii*), *HolInt* (*Holoptelea integrifolia*), and *TecGra* (*Tectona grandis*).

Tree volume estimation was performed using a hybrid PSR-QSM approach. Volumes were computed for plots with an average area of 706.8 m² (15 m radius), except for plots 2, 4, and 12, which were limited to 500 m² (12.6 m radius) due to topographic constraints. The estimated total volume ranged from 3.78 m^3^ (Plot 4) to 21.47 m^3^ (Plot 6), as illustrated in Fig. 12.

**Fig. 13:**
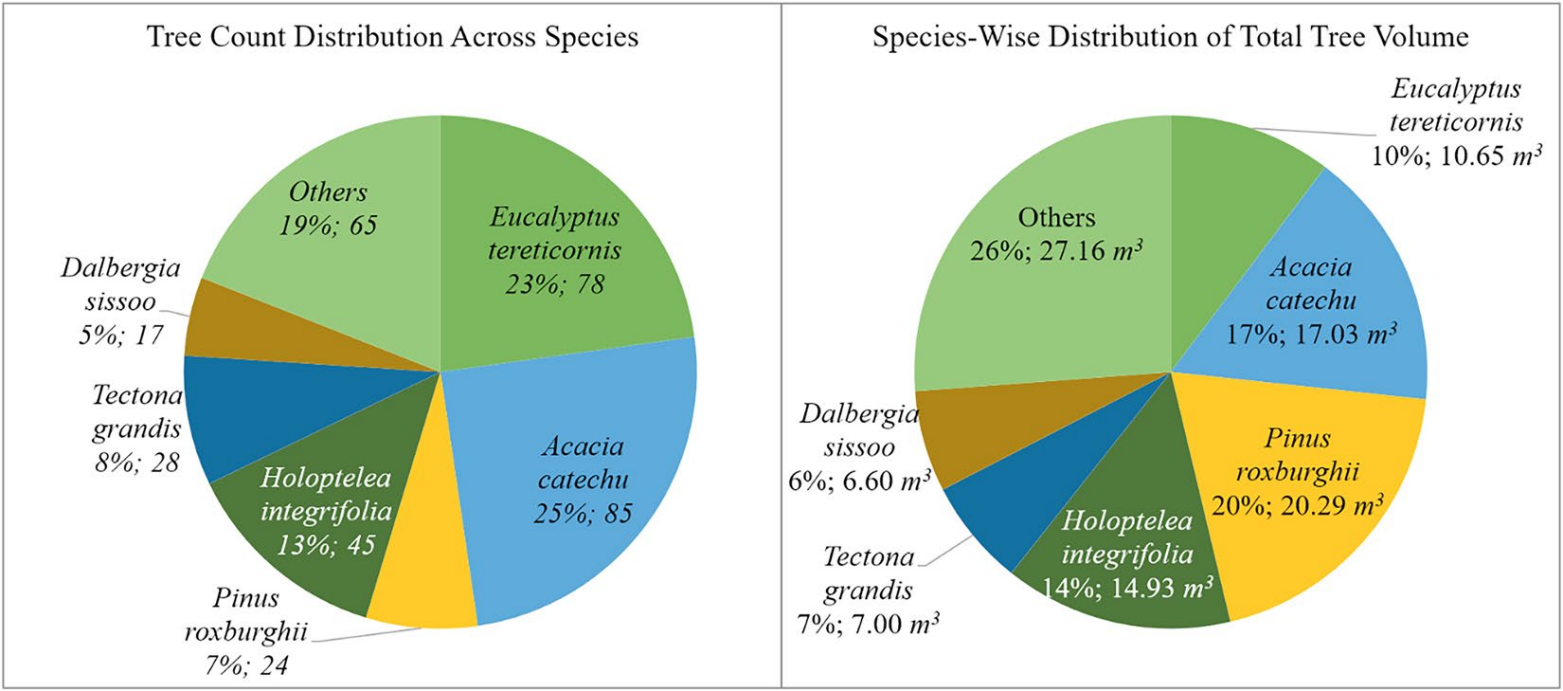
Species-wise distribution of tree count and total volume across all 12 plots included in the study. Both percentages and absolute values are provided, with total number of trees shown in the tree count distribution and total volume in cubic meters shown in the volume distribution.

Species-wise distribution, presented in Fig. 13, revealed that *Eucalyptus tereticornis* constituted 23% of the total tree count but contributed only 10% of the total volume, likely due to its younger age. Conversely, *Pinus roxburghii* accounted for only 7% of the trees but contributed 20% of the total volume, highlighting its higher per-tree biomass.

### Wood-leaf separation

Wood-leaf separation was carried out using four algorithms: LeWoS, TLSeparation, CANUPO, and RF. This analysis expanded upon the findings of Ali *et al*.^15^, who globally benchmarked geometric-based leaf-filtering algorithms using datasets from various regions. Our work applied these algorithms to a larger, regionally specific dataset, thereby validating their findings and assessing our dataset’s quality.

Table 6 summarises the overall accuracy (OA) achieved by the four algorithms on our dataset, alongside the OA reported in Ali *et al*.^15^ using similar parameter settings.

**Table 6:** Comparison of the Overall Accuracy (OA) achieved on our dataset with the OA reported in Ali *et al*.^15^ for four wood-leaf separation algorithms.

Algorithm	OA in Our Dataset	OA Reported in Ali *et al*.^15^
LeWoS	0.83	0.90
TLSeparation	0.76	0.81
CANUPO	0.88	0.89
RF	0.90	0.95

The OA achieved in our dataset is slightly lower than that reported in Ali *et al*.^15^, likely due to differences in tree characteristics in our dataset. Nonetheless, the observed performance trends were consistent with their findings: the RF algorithm demonstrated the highest accuracy, while TLSeparation performed the worst among the four.

## Usage Notes

The LiDAR dataset is openly available via LiDAVerse and Zenodo^51^ in accordance with repository policy. It is optimized for direct application in areas such as individual tree segmentation, wood-leaf classification, morphological modelling, and biomass estimation. Researchers wishing to process and analyse the dataset can use a range of available software tools. Open-source options such as CloudCompare^46^, FugroViewer^52^, LAStools^53^, OPALS^54^, CompuTree^55^, DendroCloud^56^, and the lidR^57^ library in R provide a variety of capabilities for point cloud visualisation and processing. Additionally, commercial software such as LiDAR360^45^ can be used for more advanced analyses. For specific tasks, there are several algorithms designed for specialised processing, including leaf-wood separation algorithms utilising spectral and geometric features like LeWoS^16^, TLSeparation^58^, and FSCT^59^, etc. Some commonly used geometry-based leaf-filtering algorithms are benchmarked in Ali *et al*.^15^. Moreover, for tree reconstruction and volume extraction, several models like TreeQSM^48^, AdTree^60^, AdQSM^14^, PypeTree^61^, SimpleForest^62^, and PlantScan3D^63^ can be employed to process the leaf-filtered TLS point clouds and derive tree volumes.

This dataset represents one of the first open-access forest LiDAR datasets from India, providing a valuable resource for the region’s unique forest ecosystems, which have not been widely represented in existing global datasets. Owing to the unique species composition and forest structure of Indian tropical and subtropical forests, most existing models trained on non-Indian datasets may not perform reliably here. As such, this dataset can serve as a benchmark and testing resource for various applications, including the development and testing of tree models, extraction of morphological parameters, and the advancement of tree segmentation, biomass estimation, and species classification algorithms. Furthermore, this dataset has significant potential for use as a reference in the training and validation of upcoming spaceborne remote sensing products such as NASA-ISRO’s NISAR^64^ and ESA’s BIOMASS^65^, helping to improve the accuracy and applicability of these tools in tropical and subtropical forest regions.

## Supplementary Information

Supplements

## Data Availability

The full dataset generated in this study is openly available via the Zenodo repository (10.5281/zenodo.15362444). For long-term discoverability, the dataset is also mirrored on the LiDAR point cloud repository, LiDAVerse (www.LiDAVerse.com). Both repositories provide identical versions of the data, ensuring transparency, reproducibility, and ease of reuse.

## Code availability

All code used for data processing, wood-leaf classification, feature extraction, and tree volume estimation is openly available on GitHub at https://github.com/moonis-ali/Dataset.
