# Supplementary material for: Terrestrial and Airborne Laser Scanning Dataset of Trees in the Shivalik Range, India with Field Measurements and Leaf–Wood Classifications
Source: Sci Data. 2026 Feb 11;13:420. doi: 10.1038/s41597-026-06674-w (PMC13004935; doi:10.1038/s41597-026-06674-w)
Supplement: Supplementary file 1 — Supplements [file 41597_2026_6674_MOESM1_ESM.docx]

**Table S1.** Summary of tree species, number of trees, TLS-derived height ranges, and total tree volume for each plot. The volume values represent estimates obtained using the hybrid PSR-QSM approach. Note that the data presented includes only those trees with a DBH greater than 10 cm.

| Plot ID | Species | Abbreviation | No. of Trees | TLS-derived Height Range (m) | Total Volume (m^3^) |
| --- | --- | --- | --- | --- | --- |
| Plot 01 | *Eucalyptus tereticornis* | EucTer | 27 | 12.0-19.4 | 4.41 |
| Plot 02 | *Acacia catechu* | AcaCat | 20 | 3.8-12.7 | 4.82 |
| Plot 03 | *Eucalyptus tereticornis* | EucTer | 35 | 3.3-18.3 | 3.99 |
| Plot 04 | *Acacia catechu* | AcaCat | 5 | 5.5-9.9 | 0.57 |
|  | *Chimbad* | Chimbad | 2 | 5.9-6.4 | 0.14 |
|  | *Toona ciliata* | TooCil | 3 | 7.0-8.3 | 0.22 |
|  | *Terminalia bellirica* | TerBel | 1 | 7.4 | 0.16 |
|  | *Grewia oppositifolia* | GreOpp | 4 | 4.1-8.4 | 0.44 |
|  | *Eucalyptus tereticornis* | EucTer | 5 | 9.6-10.2 | 0.51 |
|  | *Syzygium cumini* | SyzCum | 1 | 9.9 | 0.31 |
|  | *Psidium guajava* | PsiGua | 5 | 3.9-7.3 | 0.58 |
| Plot 05 | *Lannea coromandelica* | LanCor | 3 | 7.9-17.2 | 6.20 |
|  | *Delonix regia* | DelReg | 7 | 8.0-14.1 | 3.30 |
|  | *Ficus racemosa* | FicRac | 4 | 5.6-15.7 | 6.30 |
|  | *Phoenix sylvestris* | PhoSyl | 3 | 5.9-12.2 | 7.52 |
|  | *Flacourtia indica* | FlaInd | 1 | 11.4 | 0.71 |
|  | *Toona ciliata* | TooCil | 1 | 9.7 | 0.30 |
|  | *Mallotus philippinensis* | MalPhi | 3 | 6.3-8.9 | 0.31 |
|  | *Chukrasia tabularis* | ChuTab | 1 | 8.2 | 0.07 |
| Plot 06 | *Pinus roxburghii* | PinRox | 17 | 6.9-18.6 | 16.93 |
| Plot 07 | *Pinus roxburghii* | PinRox | 7 | 13.8-19.1 | 3.36 |
|  | *Acacia catechu* | AcaCat | 2 | 8.3-10.7 | 0.95 |
|  | *Bombax ceiba* | BomCei | 1 | 10.7 | 0.35 |
|  | *Terminalia bellirica* | TerBel | 1 | 12.5 | 0.26 |
|  | *Grewia oppositifolia* | GreOpp | 4 | 5.3-8.7 | 0.57 |
|  | *Holoptelea integrifolia* | HolInt | 1 | 16.9 | 1.50 |
|  | *Lannea coromandelica* | LanCor | 2 | 7.0-10.2 | 0.61 |
|  | *Cassia fistula* | CasFis | 1 | 9.9 | 0.23 |
|  | *Albizia lebbeck* | AlbLeb | 1 | 11.8 | 0.82 |
| Plot 08 | *Holoptelea integrifolia* | HolInt | 29 | 6.2-12.5 | 11.13 |
|  | *Acacia catechu* | AcaCat | 2 | 5.7-9.8 | 0.63 |
| Plot 09 | *Acacia catechu* | AcaCat | 4 | 7.1-10.3 | 0.42 |
|  | *Lannea coromandelica* | LanCor | 1 | 11.7 | 1.31 |
|  | *Tectona grandis* | TecGra | 28 | 8.6-16.3 | 7.01 |
| Plot_10 | *Eucalyptus tereticornis* | EucTer | 11 | 8.7-13.7 | 1.74 |
|  | *Acacia catechu* | AcaCat | 2 | 8.6-8.7 | 1.85 |
|  | *Flacourtia indica* | FlaInd | 1 | 7.4 | 0.98 |
|  | *Dalbergia sissoo* | DalSis | 17 | 8-16.9 | 6.60 |
|  | *Terminalia alata* | TerAla | 1 | 6.4 | 0.19 |
| Plot 11 | *Acacia catechu* | AcaCat | 24 | 3.0-13.4 | 4.88 |
|  | *Lannea coromandelica* | LanCor | 3 | 6.3-10.5 | 0.65 |
|  | *Holoptelea integrifolia* | HolInt | 6 | 7.7-10.2 | 0.71 |
|  | *Flacourtia indica* | FlaInd | 1 | 6.7 | 0.16 |
|  | *Terminalia arjuna* | TerArj | 1 | 8.5 | 0.12 |
|  | *Azadirachta indica* | AzaInd | 1 | 13.4 | 1.41 |
| Plot 12 | *Acacia catechu* | AcaCat | 26 | 3.2-10.4 | 2.92 |
|  | *Holoptelea integrifolia* | HolInt | 9 | 7.3-12.3 | 1.59 |
|  | *Flacourtia indica* | FlaInd | 1 | 3.1 | 0.04 |
|  | *Lannea coromandelica* | LanCor | 3 | 5.6-9.1 | 0.64 |
|  | *Salvadora oleoides* | SalOle | 3 | 5.9-8.0 | 0.19 |

**
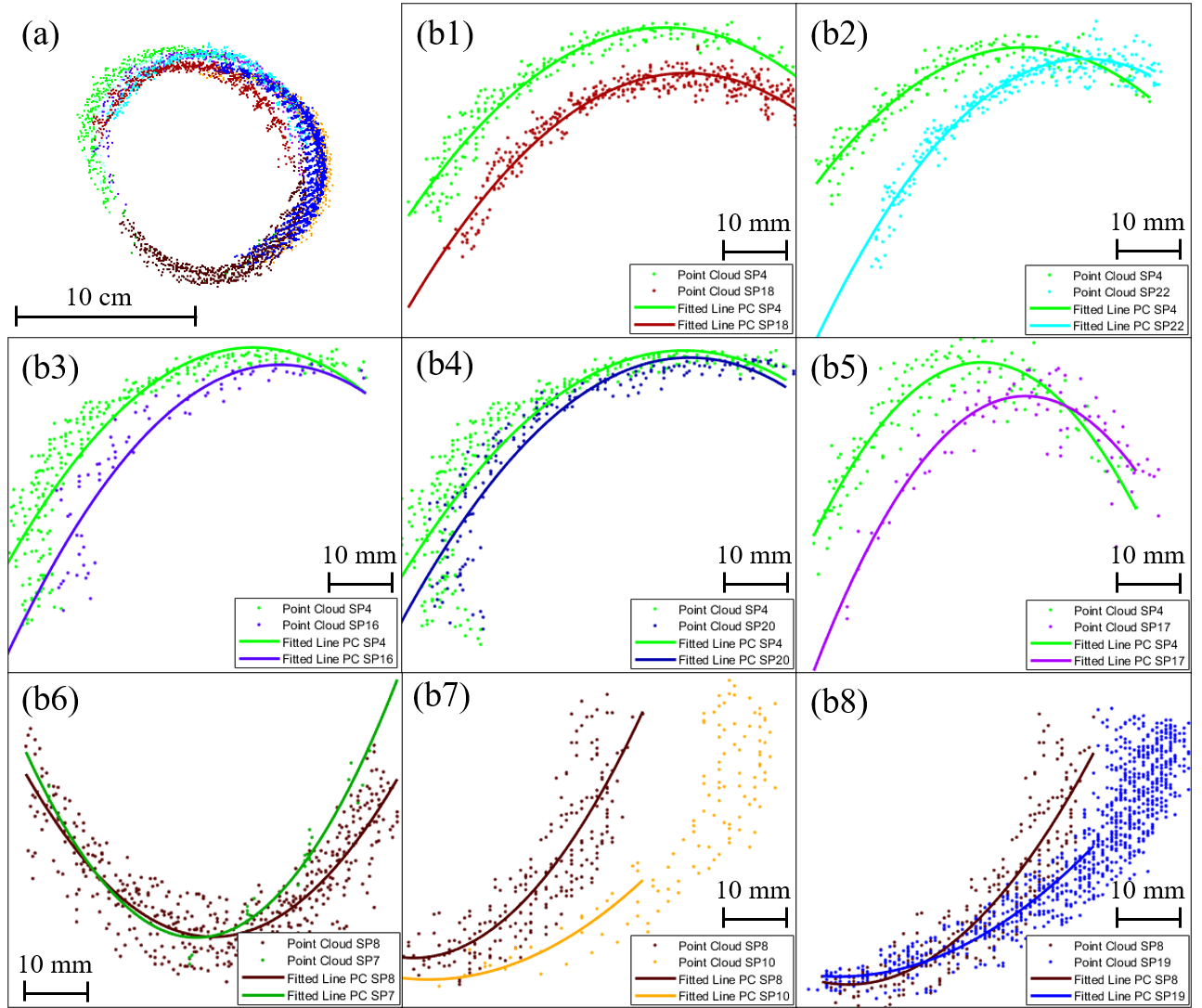
**

**Figure S1.** Horizontal registration accuracy assessment: (a) Segmented point cloud section (0.2 m to 0.4 m along the trunk from the base), with each colour representing point cloud from different scan positions. (b1–b8) Quadratic curve fitting applied to the point clouds from different stations to evaluate horizontal deviations. The horizontal axis represents X-coordinates, and the vertical axis represents Y-coordinates. SP: Scan Position; PC: Point Cloud.

**
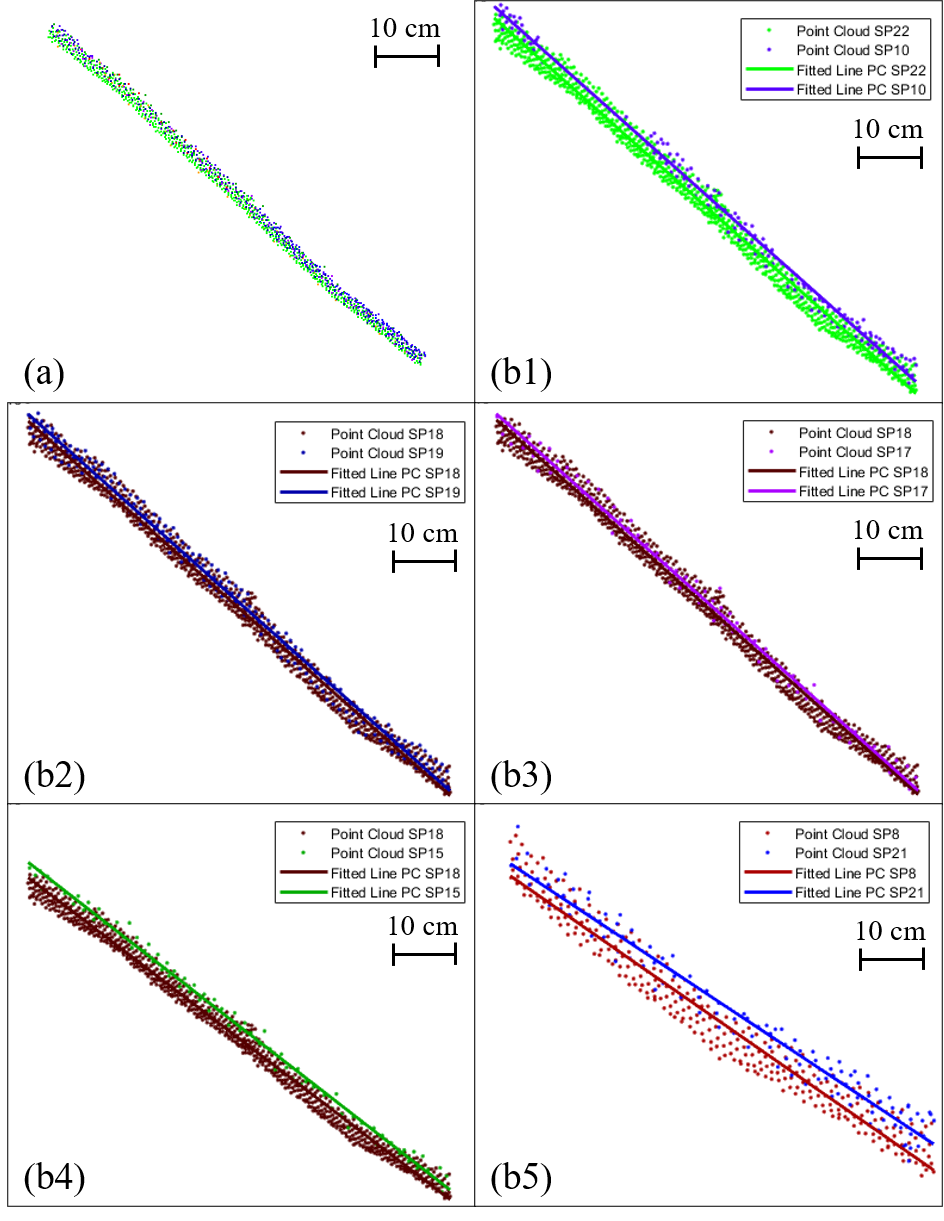
**

**Figure S2.** Vertical registration accuracy assessment: (a) Segmented second-order branch projected onto the vertical plane, with each colour representing point cloud from different scan positions. (b1–b8) Linear fitting applied to pairs of point clouds from different stations to evaluate vertical deviations. The horizontal axis represents X-coordinates, and the vertical axis represents Z-coordinates*.* SP: Scan Position; PC: Point Cloud.
